# Evaluation of Adaptive Feedback in a Smartphone-Based Game on Health Care Providers’ Learning Gain: Randomized Controlled Trial

**DOI:** 10.2196/17100

**Published:** 2020-07-06

**Authors:** Timothy Tuti, Niall Winters, Hilary Edgcombe, Naomi Muinga, Conrad Wanyama, Mike English, Chris Paton

**Affiliations:** 1 Kellogg College University of Oxford Oxford United Kingdom; 2 KEMRI-Wellcome Trust Research Programme Nairobi Kenya; 3 Department of Education University of Oxford Oxford United Kingdom; 4 Nuffield Division of Anaesthetics University of Oxford Oxford United Kingdom; 5 Nuffield Department of Medicine University of Oxford Oxford United Kingdom

**Keywords:** neonatal mortality, education, emergency medical services, global health, smartphone, feedback, health workforce, developing countries, mobile phone

## Abstract

**Background:**

Although smartphone-based emergency care training is more affordable than traditional avenues of training, it is still in its infancy, remains poorly implemented, and its current implementation modes tend to be invariant to the evolving learning needs of the intended users. In resource-limited settings, the use of such platforms coupled with gamified approaches remains largely unexplored, despite the lack of traditional training opportunities, and high mortality rates in these settings.

**Objective:**

The primary aim of this randomized experiment is to determine the effectiveness of offering adaptive versus standard feedback, on the learning gains of clinicians, through the use of a smartphone-based game that assessed their management of a simulated medical emergency. A secondary aim is to examine the effects of learner characteristics and learning spacing with repeated use of the game on the secondary outcome of individualized normalized learning gain.

**Methods:**

The experiment is aimed at clinicians who provide bedside neonatal care in low-income settings. Data were captured through an Android app installed on the study participants’ personal phones. The intervention, which was based on successful attempts at a learning task, included adaptive feedback provided within the app to the experimental arm, whereas the control arm received standardized feedback. The primary end point was completion of the second learning session. Of the 572 participants enrolled between February 2019 and July 2019, 247 (43.2%) reached the primary end point. The primary outcome was standardized relative change in learning gains between the study arms as measured by the Morris G effect size. The secondary outcomes were the participants individualized normalized learning gains.

**Results:**

The effect of adaptive feedback on care providers’ learning gain was found to be g=0.09 (95% CI −0.31 to 0.46; *P*=.47). In exploratory analysis, using normalized learning gains, when subject-treatment interaction and differential time effect was controlled for, this effect increased significantly to 0.644 (95% CI 0.35 to 0.94; *P*<.001) with immediate repetition, which is a moderate learning effect, but reduced significantly by 0.28 after a week. The overall learning change from the app use in both arms was large and may have obscured a direct effect of feedback.

**Conclusions:**

There is a considerable learning gain between the first two rounds of learning with both forms of feedback and a small added benefit of adaptive feedback after controlling for learner differences. We suggest that linking the adaptive feedback provided to care providers to how they space their repeat learning session(s) may yield higher learning gains. Future work might explore in more depth the feedback content, in particular whether or not explanatory feedback (why answers were wrong) enhances learning more than reflective feedback (information about what the right answers are).

**Trial Registration:**

Pan African Clinical Trial Registry (PACTR) 201901783811130; https://pactr.samrc.ac.za/TrialDisplay.aspx?TrialID=5836

**International Registered Report Identifier (IRRID):**

RR2-10.2196/13034

## Introduction

### Background

In low-income regions such as sub-Saharan Africa (SSA), the need for health workers to provide care by themselves is more frequent than in middle- and high-income settings, and it can be associated with negative neonatal outcomes [1]. Of the estimated 2.9 million neonatal deaths each year globally, SSA has the highest overall risk of death within the first 24 hours of life, accounting for 37% of global neonatal deaths [1]. Severe workforce shortages, coupled with the skill imbalance and maldistribution of the health workforce, and a lack of training opportunities [1,2] are likely to be key contributors to this high mortality rate. Additional training is needed to better prepare health care providers in these regions to provide effective emergency pediatric and neonatal care [3,4]. However, face-to-face training costs between US $80 and US $300 per person per day and is difficult to deliver at scale [5]. Only a small fraction of trained health providers with the basic requisite skills training for new-born resuscitation are usually found in these regions [6,7]. Therefore, new strategies are required to improve training access for over 1 million health providers across SSA. Any such approach needs to be updated efficiently in real-time as guidelines change in light of new evidence and (ideally) capture data on the number of health workers that are able to train within a certain time period [8,9].

There is little evidence about the implementation of digital learning interventions that are relevant to the context of low-income settings, that take into account health workers’ initial and continuing clinical training needs, and that adapt learning content in the light of skill mastery and performance as learners continue to develop knowledge [9-15]. In nonclinical, high-resource contexts, adaptive instructional support has been shown to significantly outperform trainer-led large-group instruction, nonadaptive computer-based instruction, and paper-based instruction in producing learning gains [4,10,16,17]. Within low-resource contexts, investigation of learner models (cognitive models that try to model observed student learning behaviors) needed to support such tailored instructional approaches in clinical training settings is justified [10,11].

Health care training apps that have been developed to date and the approaches used can broadly be divided into two categories: those that simply replicate existing teaching strategies “on a screen,” for example, by providing questions and answers for exam practice or displaying textbook graphics, that is, the “drill-and-practice” pedagogical approach, and others, that take advantage of features specific to digital devices, examples of which include the ability to respond with different pathways to user choices or the use of animations with which the user can interact, that is, a more learner-centered pedagogical approach [9]. Serious games, which are games with a specific, applied purpose (other than entertainment) that can be played on mobile phones, are one such way of providing training with the potential to affect health outcomes [18]. The rationale for using serious games is that, in a similar way to “first-person” computer games, emergency care training should enable health workers to follow highly structured pathways (such as clinical care algorithms) with pieces of information (cues) sought at each step that determine the correct actions to perform. With both clinical training and performance in computer games, executing cue-response sequences perfectly, rapidly, and automatically, with minimal help (eg, from corrective feedback and hints), demonstrates mastery. This type of mastery has been shown to support effective clinical care delivery, but the required frequency of rehearsal in this approach is difficult and expensive to maintain for face-to-face training [19]. By using a serious gaming approach, users may be more motivated to repeatedly play the serious game, using incentives such as rewards, increasing difficulty, and scores—techniques that have been successfully used to encourage repeated gameplay in nonserious computer games. There is a scarcity of evidence from the evaluation and assessment of serious gaming approaches in smartphones for health care training in low-income settings. Addressing this need was highlighted in the most recent systematic review in this area [15,20].

The Life-Saving Instruction for Emergencies (LIFE) project uses a smartphone-based serious game approach initially to provide training in the care of very sick newborns and children. The app extends the scenario-based Emergency Triage, Assessment, and Treatment Plus admission care training (ETAT+) training model [21,22] by incorporating more learner-centric intervention approaches. The aim of ETAT+ is to familiarize health care providers with clinical guidelines and the necessary knowledge and skills for triaging all sick children when they arrive at a health facility into those with emergency signs, with priority signs, or nonurgent cases and provide emergency treatment for those with life-threatening conditions [23]. The ETAT+ teaching model uses a face-to-face model to train health care workers in Africa and Southeast Asia and is explained in detail elsewhere [21-25].

Given the contextual challenges of enhancing health care providers’ learning settings, such as SSA, LIFE is designed to develop health care providers’ self-regulating learning (SRL) of ETAT+ content independent of any classroom or face-to-face tutoring facilitation. The potential for utilizing the digital and reusable nature of interventions such as LIFE to adapt the way health care professionals learn and receive feedback on their performance remains underexplored in SSA. Such personalization of learning could be used to maximize learning outcomes and to develop learners’ skills [14,15]. The ubiquitous nature of smartphones as experimental tools offers access to a wider pool of study participants [26] and can minimize the cost of implementing, evaluating, and scaling educational platforms such as LIFE in a resource-constrained context [9]. Smartphones have also been shown to raise learners’ interest in learning interventions [27].

SRL is a weakly recursively process that is facilitated by feedback at each stage [28-30] supporting learners’ metacognitive regulation of learning strategies to help them regulate resources and emotions while learning [29]. In addition to regulation, metacognition consists of declarative knowledge and deals with the interplay between knowledge of one's abilities to perform tasks, the nature of the learning task, and the strategies one can employ to successfully perform the task [29]. It is theorized to have a limited capacity that renders the learners prone to making errors in complex or time-limited learning tasks (eg, delivery of emergency care in newborns) [31]. In the presence of such performance errors (eg, slipping or guessing) due to cognition’s limited capacity [32], to guide SRL, a key objective is to infer the knowledge of the learning task being tutored. Feedback is posited to enhance learning when “...it provides further information to correct or modify action through the construction and activation of more appropriate [action sequences] ...” [33]. The information provided ought to move the learner to a deeper understanding of the learning task [34]. However, more feedback does not always equate to better learning: “...The amount of information given to the student must be what the student can use, rather than the amount the [tutor] may wish to give...,” especially in light of the limited capacity of cognition [32,34-36]. Feedback that is too elaborate is more likely to produce cognitive overload. On the other hand, if it has low specificity, it is more likely to be perceived by learners as useless [32,37]. Effective feedback is posited to be specific but not too elaborate and presented in manageable units [37,38]. Its timing, specificity, frequency, and type have varying effects in enhancing learning [36]. In the absence of flexibility in determining the instruction challenge level or stratifying learning pathways within the LIFE project (due to the efforts to standardize delivery of clinical guidelines training content), feedback remains the most promising intervenable theory-aligned concept for enhancing SRL using smartphones in settings such as SSA.

### Objectives

The primary objective of this randomized experiment was to investigate whether adaptive individualized feedback is superior to standardized feedback in mobile smartphone-based emergency neonatal care training. We hypothesized that health care providers randomized to receive adaptive feedback would have significantly higher learning gains than those randomized to receive standardized feedback. The secondary objective was to investigate learning gain in general and how learning gains when using LIFE are modified by health care providers’ characteristics and how they space their learning.

## Methods

### Ethical Approval

The breakdown of the methods and analysis plan for this experiment are described in the published protocol for this study [39] and was approved by the Kenya Medical Research Institute’s (KEMRI) Scientific and Ethical Review Committee (#3444) and the Central University Research and Ethics Committee of Oxford University (#ED-CIA-18-106). It follows the Consolidated Standards of Reporting Trials guidelines for reporting randomized experiments [40].

### Study Design

The study was a parallel-group, double-blinded, randomized experimental design with an allocation ratio of 1:1. The participants were randomized to receive the intervention or to be in the control group when they launched the training app for the first time on their individual smartphone devices.

### Eligibility Criteria

The participants were health care providers from any professional cadre involved in providing bedside patient care, who were either undergoing training (eg, students), or actively providing nursing, clinical or medical care. Health care providers who had retired from clinical practice, who practiced in high-income settings, or participants who were not health care providers were excluded from the study.

### Study Setting and Recruitment

This study was confined to participants from low-income countries who stand to benefit from training in the management of pediatric emergencies (Multimedia Appendix 1). The distribution of the LIFE smartphone app was through the Google Play Store, with initial efforts directed toward face-to-face recruitment of participants in Kenya (more details are provided in the study protocol [39]). The recruitment of study participants endeavored to promote voluntary self-enrolment and used snowballing and purposive sampling strategy [41], which have been explained in detail in the study protocol [39].

### The Intervention

The intervention in this study was the adaptive differentiated immediate feedback provided while learning through a smartphone-based serious gaming app. The content to be learnt was based on a neonatal resuscitation guideline course that is already offered in nine low-income countries [21,22,24]. The smartphone-based app was publicly available on the Google Play Store, where it was downloadable and installable to compatible Android-based smartphones. All study participants received a link to the mobile app hosted on the Google Play Store. The LIFE app was designed to target Android’s SDK19 as the minimum version of Android supported as at February 2019 (which targets 100% of Android devices) [42]. The smartphone app had already undergone alpha and beta testing on a pilot cohort of health care providers’ smartphones from Kenya since October 2017 to ensure stability and reliability of its functioning on different mobile phones. More details of the intervention are provided in the Multimedia Appendix 2 [43-45].

The number of standardized feedback levels was determined by global health academics and expert medical trainers involved in ETAT+ training in SSA in consultations with the relevant medical professional bodies accrediting continuous professional development. The adaptive immediate feedback provided to the experimental group participants was designed to arouse meaningful reflective learning from continuous interaction between the learners and the smartphone-based training [46,47]. As didactic-procedural form of feedback [48] aligned to our theoretical framing, it was designed to force the health care provider to contemplate over the incorrect care provision choices they provided in their failed attempt (eg, “*Some* of the selected actions are not *appropriate* at this *stage*” focuses on the number of wrong choices and their placement within the clinical care-giving pathway and is meant to force reflection as to which stage they are most appropriate). This feedback was provided to the experiment arm after each incorrect attempt at a learning task with three cascading detail levels based on the predicted probability that the learner’s next attempt was going to be correct. The modeling approach to support the data collected from 187 health care providers during the beta testing phase and is briefly described in the Multimedia Appendix 2 and explained in detail elsewhere [49]. The wording of the feedback provided was dependent on the number of incorrect choices the learner had selected and the actual incorrect choices themselves. This is illustrated in Multimedia Appendices 3-6. The control group study participants received standardized nonadapted immediate feedback after each incorrect attempt at a learning task, with the feedback on the first incorrect attempt asking the learner to retry (level 0, Multimedia Appendix 6) and the feedback on the second attempt giving a detailed explanation of the correct choices to select (level 2, Multimedia Appendix 6). Using multidimensional model of personalization by Holmes et al [50], in this experiment, the adaptive mechanism targets the personalization of how feedback is to be presented and when it is to be introduced*.*

The LIFE app was both the learning and the measurement tool. The learning task is synonymous with the quiz as learning is designed to be embedded in the formative evaluation. At the end of a successful completion of a learning session, the platform provided a performance score based on whether each learner’s response to the learning tasks was correct on the first attempt. For this study, a learning session was conceptualized as every unique initiation (ie, iteration) of the neonatal resuscitation learning scenario training round on the LIFE smartphone app (illustrated in Scenario A in Multimedia Appendix 7).

### Outcomes

The primary end point for both arms of the experiment was the completion of two learning sessions using LIFE, the first session being treated as pretest and the second session being treated as posttest. Both scores were converted into percentages. From the pre-post scores, the study’s main comparative outcome was the learning effect size (g) [51], with the formula for its calculation provided in this study’s protocol [39] and provided in the Multimedia Appendix 2. This effect size, also referred to as *Morris G* [51], represents the mean difference between the relative change within the study arms. From education literature [16,52,53], effect sizes of approximately 0.2, 0.5, and 0.8 are considered as small, moderate, and large effect sizes, respectively. These thresholds represent the magnitude of the effect and reflect our assumption that a statistically significant result is not necessarily important or meaningful. For example, for an effect size of approximately 0.2, the difference between the study groups is trivial even if it is statistically significant [52,54].

In addition to randomization, which eliminates or at least dramatically reduces biases influencing this study’s primary outcome, the calculation of this study’s primary outcome (1) is robust in managing preexisting knowledge differences among learners, (2) allows for the estimation of the intervention effect even when experimental and control groups are nonequivalent, and (3) considers the variances of both pretest and posttest scores. This contrasts with other forms of effect size calculation such as Hedges G and Becker D, which only use pretest or pooled variances [51]. In this model, the pretest and posttest variances were assumed to be homogeneous. The secondary outcome considered was the individualized learning gain of the study participants, defined as the relative change in performance score of health care providers divided by the maximum score they could have improved upon [55]. This was calculated from the performance scores from learning sessions following each other, with the performance from the first session treated as pretest score and the one from the last session treated as a posttest score. The formula for this calculation is provided in this study’s protocol [39].

### Participant Timeline

Enrolment of study participants began on February 1, 2019, and continued up to July 31, 2019. This study’s rollout of LIFE’s intervention was based on implementation study principles and outcomes [56] and was informed by self-regulated, self-directed learning [28,31,57]. It sought to understand and work within real-world conditions, rather than trying to control for adoption, acceptability, coverage, and sustainability conditions or to remove their influence on the study outcome [56]. Subsequently, no training sessions were planned for the study participants. Although LIFE is designed for low-income contexts, there was no limit set by geographical coverage for health care providers who might be interested in undertaking this self-directed training; anyone could download the app. However, we only analyzed health care providers from low-income countries. Participants without any geographic location data (due to refusing to grant the LIFE app the required Android permissions) were assumed to be from developing countries, given that our recruitment efforts were directed toward professional groups in these countries.

### Sample Size Calculation

Similar interventions in other subject domains have been found to have a mean effect size of between 0.22 (95% CI 0.16 to 0.27) to 0.70 (95% CI −0.08 to 1.49) [16,58]. Drawing from these studies, to detect an effect size of 0.22 with a two-sided 5% significance level and a power of 80%, a sample size of 83 participants per group who reach the primary end point of the study was necessary. A sample size calculation for a one-way analysis of variance, together with one-sample and paired-sample *t* test analysis using the same effect, power, and significance parameters produced the same required sample size of 166 participants. The sample size calculation formula is provided in this study’s protocol [39].

On the basis of the alpha tests of the LIFE smartphone app, we assumed a 50% dropout rate of study participants, with dropout defined as the incomplete or single use of the LIFE smartphone app, and planned to recruit at least 332 participants to account for this dropout rate. To encourage repeated usage of LIFE, all participants received email reminders from the time they were enrolled in the study once every 2 weeks, and this was suspended after they had received three reminders. From interviews of the study participants in the alpha and beta phases of the LIFE app development, those from lower clinical cadres tended to characterize the cost of phone data charge necessary for downloading the 231 MB smartphone app as too high. To mitigate the burden of saddling participants with this extra cost to their personal finances due to participating in the study, they could request for reimbursement of costs within a few weeks of using the LIFE smartphone app.

Demographic data was collected within the app in its initial use by study participants at the end of the first learning session if they consented. This was because demographic data were deemed more sensitive than trace play data and therefore required additional consent as per the ethical approval. This meant that for learners who dropped out before completing the first session, or chose not to fill-in those data, no demographic data were collected. From these data, statistical analyses were conducted to evaluate whether there was any systematic bias in the attrition of study participants. Study variables used in this analysis were study groups, exposure to previous neonatal training, clinical cadre, age, and level of experience. This was because given the differences in training pathways for clinical cadres and length of practice, level of expertise might produce differences in the effectiveness of the learning intervention [59]. In addition, age was included to evaluate whether it was associated with the pattern and effectiveness of smartphone-based learning given its novel nature requiring digital acuity [60]. We judged sex not to be theoretically influenced by the socio-cognitive framing of this experiment’s research questions.

### Randomization

For allocation of the participants, an in-app algorithm randomly generated a value between 0 and 3 when the smartphone app was launched for the first time. If the value was either 0 or 1, it was recoded to 0, otherwise recoded as 1. This algorithm was implemented using a randomization routine provided by the game engine for development reasons, which we assumed to be reliable [61]. The algorithm determined whether a participant was allocated to the control (if the recoded value was 0) or the experimental group (if the recoded value was 1). It also blinded both the study participants and staff to the allocation of participants to groups during the experiment, but not at the analysis stage. Sequence generation for random allocation was a computerized procedure pegged on a single instance (ie, smartphone app installation instance) that mimicked a coin-tossing procedure. Therefore, using permuted blocks of random sizes to assign participants to either the control or experimental group was not possible and therefore not implemented.

### Statistical Methods and Analyses

For the primary end point, we used the Morris G effect size to analyze the differences between study arms of relative change in scores within the arm, as described in detail in the study protocol [39]. This was assessed after the second round of completing the training scenario through the smartphone app, with each round’s performance score recorded. Secondary analysis was conducted using regression analysis, with the dependent variable being the normalized learning gain and the independent variables being health care providers’ demographic characteristics and the game play characteristics (eg, spacing of repeat learning session, amount of time spent on learning task, and previous exposure to neonatal training), to evaluate their effect on learning gains.

The primary learning outcome used in this study could not be computed for study participants whose dropout was characterized by a lack of at least two complete learning sessions. Without a postbaseline assessment, “intention-to-treat” analysis could not be performed unless we imputed outcomes, which tends to produce biased estimates [62]. Therefore, we did not conduct an intention-to-treat analysis. However, dropout numbers are reported in relation to those who reached the study’s primary end point, with their implications discussed considering self-regulated, self-directed learning [28,31,57].

Qualitative interviews were conducted in parallel to the experiment from a small sample (N=19) of the health care providers who participated in the study, regardless of whether they reached the primary end point or not. These interviews were used to explore health care providers perceptions of self-regulated learning that affected the contextual use of the smartphone-based learning platform. They provided a context for interpreting the observed learning outcomes from this study and will be reported separately.

### Data Management

The primary data collected from the study participants’ Android smartphone app are held on their devices with a back-up copy synchronized to *Google Firebase,* a secure distributed cloud-based database server, after being transmitted in an encrypted format. Data collected after the experiment were stored on encrypted password-protected USB devices and transferred to secure password-protected servers in Kenya and Oxford. Deidentified data were shared with study staff based at the research institutions listed on the ethical approval forms.

### Informed Consent

Individual participant consent was elicited from within the mobile app before collection of any demographic data, in addition to using explicit Android permission requests for collecting trace anonymized learning data from the app. This approach of informed consent in an app is not uncommon in medical research; it has been described in detail in a systematic review [63] as well as specifically for mobile app–based research [64]. The informed consent process also included information on the credentials and affiliations of the researchers and developers of the LIFE platform. Participants had no way of knowing whether they were receiving the “intervention of interest.”

## Results

### Study Sample

**Figure 1:**
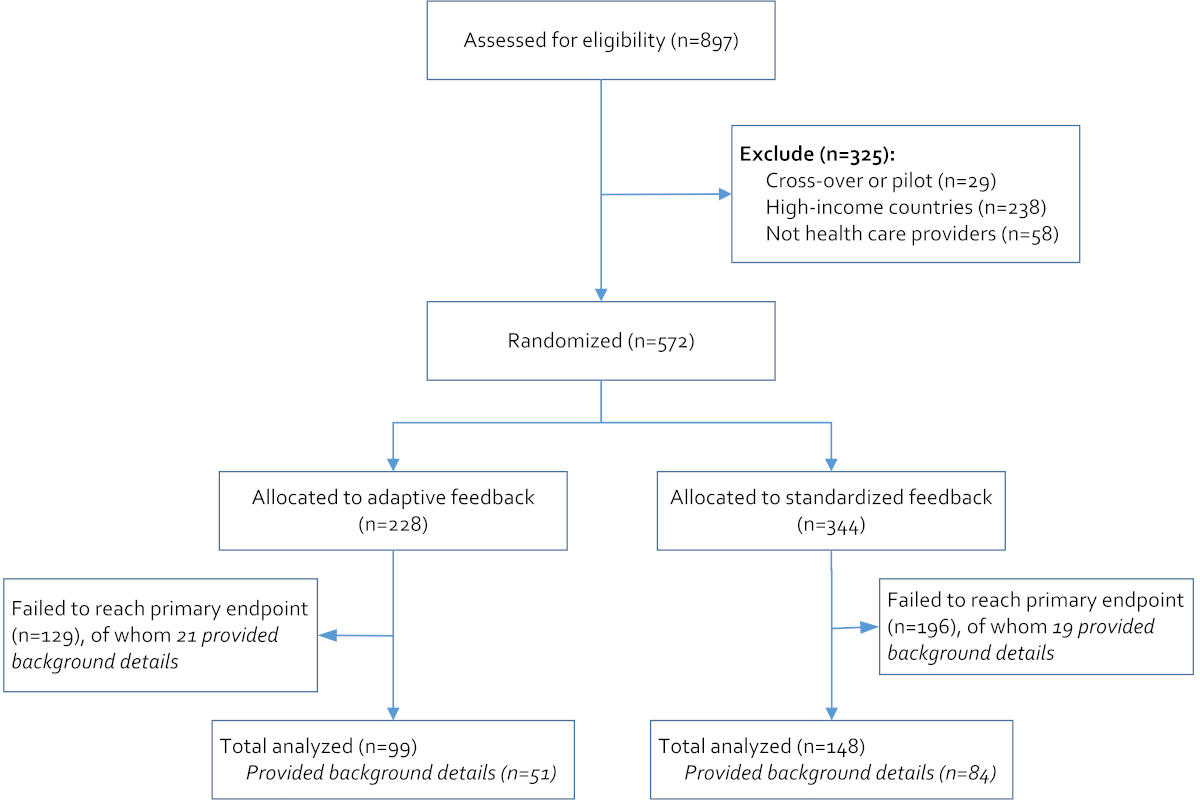
Experiment’s enrolment flowchart.

At the end of the data collection period, 572 of the enrolled 897 participants were eligible for the study and 247 (43.2%) ended up reaching the study’s primary end point (Figure 1), as expected in our published protocol [39].

It was challenging to enforce the allocation ratio because the algorithm used for randomization was not centralized and therefore unable to implement a blocking mechanism in participant to group allocation. Randomization was happening on the individual health care provider’s personal smartphone independent of how other participants were allocated and is illustrated in detail in Multimedia Appendix 8. In addition to the lack of an enforceable blocking mechanism, the software library used for the random generator function (C++ rand function) is most likely to have caused an unequal allocation ratio because it has varied efficiency across multiple smartphone devices that remain largely unknown (Multimedia Appendix 9) [61,65]. It is highly likely that this was what contributed to the observed disparity in allocation ratio. However, this did not render the allocation of study participants to the study arms nonrandom, as detailed below.

A total of 30.6% (175/572) of the enrolled participants provided their background information, and of these, 135 reached the experiment’s primary end point (Figure 1). From the difference in means test and difference in proportions test, there was no significant difference between the experimental and control groups of both those who dropped out and those who reached the study’s end point by LIFE use characteristics (Multimedia Appendix 10) or demographic characteristics (Table 1). However, the dropout rate was significant in health care providers in clinical officer cadres and those who had specialized training (Multimedia Appendix 11).

**Table 1:** Baseline characteristics of study participants where demographic data are available.

Indicator		All health care providers who provided background information (n=175)			Health care providers who reached the study’s primary end point (n=135)		
		Control (n=103)	Experiment (n=72)	*P* value^a^	Control (n=84)	Experiment (n=51)	*P* value^b^
Age (years), mean (SD)		31.2 (8.26)	29.4 (6.99)	.12	30.81 (8.42)	29.18 (7.24)	.24
Experience (years), mean (SD)		7.21 (7.05)	6.44 (7.81)	.52	6.83 (6.66)	6.46 (8.33)	.79
Sessions started, mean (SD)		3.92 (3.02)	4.22 (3.37)	.55	4.32 (3.00)	5.35 (3.39)	.08
**Clinical cadre, n (%)**							
	Doctor	37 (35.9)	30 (42)	.33	31 (37)	22 (43)	.24
	Clinical officer	17 (16.5)	10 (14)	.26	14 (17)	5 (10)	.87
	Nurse	40 (38.8)	28 (39)	.82	31 (37)	21 (41)	.31
	Other	9 (8.7)	4 (5)	.53	8 (9)	3 (6)	.77
**Clinical training level (whether completed general/specialty training and professionally registered), n (%)**							
	Specialized	23 (22.3)	11 (15)	.42	19 (23)	6 (12)	.94
	General officer	38 (36.9)	29 (40)	.72	29 (35)	21 (41)	.22
	Intern	9 (8.7)	7 (10)	.53	8 (9)	6 (12)	.34
	Student	33 (32.0)	25 (35)	.32	28 (33)	18 (35)	.41
Done Emergency Triage, Assessment, and Treatment Plus admission care training?, n (%)		60 (58.3)	47 (65)	.28	49 (58)	33 (65)	.23

^a^*P* value from difference in means between study arms within progression category.

^b^*P* value from a difference in proportions test between study arms within progression category.

The effects of attrition bias appear to be minimal, as reported in Table 1 and Multimedia Appendix 10. There is good reason to infer that the randomization generally achieved its purposes of balancing participants from different backgrounds between the intervention arms while also mitigating against selection bias despite the unexpected allocation ratio.

### Effect of Adaptive Feedback on Learning Gains Across Study Arms

The performance of the learners showed a substantive improvement of about 30% above pretest scores in both the control and experimental arms (Table 2). As the overall learning change from LIFE use was large, this may obscure a direct effect from the adaptive feedback. From Levene’s test, the assumption that the variance of the performance scores was homogenous holds true [66].

**Table 2:** Performance of learners within study arms.

Study group	N	Pretest (%), mean (SD)	Posttest (%), mean (SD)	Life-Saving Instructions for Emergencies smartphone app effect^a^	
				Correlation^b^	Effect size (95% CI)
Control group	148	56.1 (23.2)	85.8 (15.6)	0.226	1.031 (0.789-1.274)
Experiment group	99	50.4 (21.4)	81.8 (17.7)	0.335	1.272 (0.966-1.577)

^a^Effect size based on intra-individual changes in test scores accounting for correlation between the scores in a single-group pretest posttest design [51].

^b^Pearson R correlation between the scores within the study arm.

The main outcome of interest, which was the effect of adaptive feedback on learning gain, was calculated after the second round of the completed learning scenario, and its calculation is explained in detail elsewhere [39]. Adaptive feedback had an intervention effect of 0.09 (95% CI −0.31 to 0.46; *P*=.47), which is both statistically and substantively insignificant. It is noteworthy that the calculation for the effect size computed here does not treat the scores as repeated measures but as independent data. It also assumes that pretest and posttest variances are homogeneous. To minimize this bias, a correction adjustment factor of 0.998 was applied to the effect size and is explained in the protocol [39,51]. This suggests that the degree of bias in the effect size calculation was minimal given that it is a very small adjustment. The number of feedback messages provided in the experiment over the learning sessions is illustrated in Table 3.

**Table 3:** Number of feedback messages provided.

Study arm		Minimal, n (%)	Reflective, n (%)	Detailed, n (%)	All, n (%)
**Feedback messages for the 572 learners who were enrolled into the study**					
	Control	2067 (27.96)	0 (0.00)	2348 (31.76)	4415 (59.71)
	Experiment	269 (3.64)	1097 (14.84)	1613 (21.82)	2979 (40.29)
	All	2336 (31.59)	1097 (14.84)	3961 (53.57)	7394 (100.00)
**Feedback messages^a^ for the 247 learners who reached the study’s end point**					
	Control	899 (29.4)	0 (0.00)	955 (31.23)	1854 (60.63)
	Experiment	48 (1.57)	543 (17.76)	613 (20.05)	1204 (39.37)
	All	947 (30.97)	543 (17.76)	1568 (51.28)	3058 (100.00)

^a^Counts are only for the first two complete learning sessions.

### Effect of Adaptive Feedback on Individual Learning Gains

The primary outcome analysis results are likely to suffer from two forms of detection bias: subject-treatment interaction bias and differential time-effect bias [67], as illustrated in Multimedia Appendix 12. To mitigate against these biases and explore how LIFE use and learner background characteristics might affect learning gains, we conducted a secondary analysis using normalized learning gain at the individual level. This was consistent with the published protocol [39]. Details of our calculation of the normalized gain are explained elsewhere [39,55].

When considering differences in LIFE use at the individual health care provider level, learners who had a space of more than a week between subsequent use of LIFE had significantly lower normalized gains by −0.395 (95% CI −0.557 to −0.232; *P*<.001) than those who had spaced their learning to an hour or less. Any variation in spacing the self-directed use of LIFE, which was a week or less, did not produce significant changes to the normalized gains. The adaptive feedback mechanism had a significant effect on health care providers’ normalized learning gains of 0.523 (95% CI 0.345-0.702; *P*<.001). Longer time spent on learning tasks and ratio of feedback hints provided per attempt at a learning task were significantly associated with lower normalized student learning gains. Health care providers’ previous face-to-face training in ETAT+ content had no positive significant effect on their learning gain. This is illustrated in Table 4, model A.

When considering the demographic and background characteristics of the health care providers, their clinical cadre and level of practice/training had no significant effect on their learning gains, except for doctors whose learning gains were significantly higher by 0.14 (95% CI 0.016-0.265; *P*=.027). This is illustrated in model B of Table 4. Controlling for health care providers’ background characteristics significantly increased the effect of adaptive feedback on individualized normalized learning gains to 0.644 (95% CI 0.347-0.941; *P*<.001). It also improved the proportion of variance for average student normalized gain, which was explained by the independent variables in the regression model A by 18.3% (Table 4). Overall, independent variables had low multicollinearity in both variants of the regression model in Table 4, as illustrated in Multimedia Appendix 13. Both models explained 34.4%-40.7% of the variance in the normalized learning gains of the health care providers using the LIFE smartphone app to train on neonatal emergency care. However, only model B in Table 4 satisfied all the statistical modeling assumptions.

A fraction of the learners had more than two rounds of play, as illustrated in Multimedia Appendices 14-16. However, with each round of play, the numbers of health care providers dropped by around 40%-60%. This meant that fitting a longitudinal model was not feasible because the variances of more than one linear combination of time effects were close to zero (ie, singular), indicating that the model would be overfitting [68]. Furthermore, health care providers’ spacing of their learning was not standardized, and the learning iteration variable violated the sphericity assumption necessary for conducting a repeated measures analysis of variance test [69].

**Table 4:** Learning intervention effect on individual health care providers’ normalized learning gains.

Indicator		Model A^a^: all learners (n=247)						Model B^b^: learners with demographic information (n=135)					
		β (SE)		*P* value		95% CI		β (SE)		*P* value		95% CI	
Intercept		.79 (0.042)		<.001		0.707-0.872		0.851 (0.088)		<.001		0.677-1.026	
**Reference: Spacing** **≤** **1 hour**													
	Spacing ≤1 day		.027 (0.054)		.61		−0.078 to 0.133		−0.045 (0.072)		.53		−0.188 to 0.098
	Spacing ≤1 week		−0.142 (0.078)		.07		−0.294 to 0.011		−0.28 (0.097)		.005		−0.472 to −0.088
	Spacing ≤1 month		−0.395 (0.082)		<.001		−0.557 to −0.232		−0.445 (0.129)		<.001		−0.7 to −0.19
**Reference: Group=control**													
	Group=experiment		.523 (0.091)		<.001		0.345-0.702		.644 (0.15)		<.001		0.347-0.941
	Time spent on learning task		−0.09 (0.023)		<.001		−0.135 to −0.046		−0.036 (0.038)		.35		−0.11 to 0.039
	Help ratio^c^		−0.826 (0.133)		<.001		−1.087 to −0.565		−1.116 (0.219)		<.001		−1.549 to −0.683
**Reference: Done Emergency Triage, Assessment, and Treatment Plus admission care training before=no**													
	Done Emergency Triage, Assessment, and Treatment Plus admission care training before=yes		−0.013 (0.04)		.75		−0.092 to 0.066		−0.092 (0.056)		.11		−0.204 to 0.02
**Reference: Cadre=nurse**													
	Cadre=clinical officer		—^d^		—		—		0.01 (0.085)		.90		−0.15 to 0.179
	Cadre=doctor		—		—		—		0.14 (0.063)		.03		0.016-0.265
	Cadre=other		—		—		—		0.124 (0.107)		.25		−0.088 to 0.336
**Reference: Level=student**													
	Level=intern		—		—		—		0.007 (0.096)		.95		−0.184 to 0.197
	Level=general officer		—		—		—		−0.001 (0.07)		.99		−0.139 to 0.137
	Level=specialized		—		—		—		−0.045 (0.085)		.60		−0.213 to 0.123
Age (years)		—		—		—		0.022 (0.027)		.42		−0.031 to 0.075	
Experience (years)		—		—		—		−0.033 (0.031)		.30		−0.094 to 0.029	

^a^Adjusted R^2^ for model A was 0.344, and *P* value from the Breusch-Pagan test for homoscedasticity for model A was .02. Heteroskedasticity is indicated if *P* value is <.05

^b^Adjusted R^2^ for model B was 0.407, and *P* value from the Breusch-Pagan test for homoscedasticity for model B was 0.61. Heteroskedasticity is indicated if *P* value is <.05

^c^The number of hints given as a ratio of number of tries a learner had in the second single learning session.

^d^They are estimates of indicators for the corresponding column heading.

## Discussion

### Summary of Findings

This study was used to explore the effectiveness of adaptive feedback for smartphone-based training of health care workers in low-income settings, which is a largely unexplored topic of medical education in this context. We found that although there was considerable learning gain with both forms of feedback (Table 2), adaptive feedback had a weak effect of 0.09 (95% CI −0.31 to 0.46; *P*=.47), which was not statistically significant. However, when considering the background characteristics of health care providers and the various self-directed spaced learning options, and using learning gains analyzed at the individual level as opposed to the group level, adaptive feedback had an effect size of 0.644 (95% CI 0.347-0.941; *P*<.001) on student normalized learning gains with immediate repetition. Spaced learning of a week or more was associated with a significant reduction in normalized gains by 0.28. Differences in clinical cadre, level of practice/training, and previous exposure to neonatal emergency care training had no significant effect on the individual health care providers’ learning gains.

### Relation to Other Studies

This experiment differs from previous similar studies in digital education for clinical practice in that it (1) used mobile devices for delivery of digital education interventions, (2) evaluated novel educational modalities enabling simulated learning such as adaptive feedback, (3) provided essential methodological information necessary for comparability purposes, (4) reported relative learning gains as done in this study except one (rather they tended to report differences of postintervention scores in the study arms), and (5) were from low-income settings such as SSA or South-East Asia [11]. In the three studies that dealt with resuscitation identified by the systematic review by Car et al [11], only one study reported relative mean change but found no significant effect of different formulations of online content on learning gain [70], which is similar to our findings, although our primary outcome is calculated differently from theirs. In that study, a variant of individual students’ normalized gain was used, such as the secondary outcome we used in this study where we found the effect on individual student learning gains was 0.644 (95% CI 0.347-0.941; *P*<.001) when considering individual learner characteristics.

This study has addressed some of the recommendations from a recent evidence review into gamified education in health, which proposed future studies to employ the use of a rigorous experimental design to evaluate learning interventions, and include more studies from low- and middle-income countries, two underrepresented aspects of the current evidence base [15]. Even when considering interventions looking into adaptive feedback in digital education, none that we know of are in the health domain [71], making this study unique. Given that the implementation of the learning intervention was available to all clinical cadres involved with bedside care provision, who had varying levels of experience (from students to consultants), from multiple low-income countries, representing a varied mix of geographical and resource settings, the diverse population of the clinical taskforce in this experiment ensures that those who would most benefit from using the presented learning tool for training in emergency care delivery are well represented. Furthermore, as the overall learning gain from LIFE use in both study arms was large, given that the learning platform minimized elements that would not be typically available in routine app settings [56], this experiment’s findings are generalizable to emergency care training of the health care provider population from low-income countries using smartphone-based platforms.

### Implications of Findings

At the group level, the effect of adaptive feedback on health care provider learning gains was not significant, but the opposite is true when evaluated at an individual level. This might imply that the intervention effect is strongly mediated by other factors (which were external to the smartphone app), chief among them being health care providers’ individualized spacing of learning repetition. Together, with the inclusion of demographic characteristics, this increased the explainable variance of adaptive feedback on individual learning gains by 18.31%. Linking the level of adaptive feedback provided to health care providers’ individualized learning repetitions conditioned on their level of experience and clinical role might explain the stronger intervention effect at the individual learner level.

This difference between the group and individual learning gain might also imply that the decision of when to collect postintervention or repeated measures is a significant factor in determining what is effective learning intervention. Considering spaced learning, intuitively, the intervention effect becomes more reflective of a mechanism that works on optimizing the recall rate as opposed to the level of internalizing/understanding content. Despite efforts to encourage the latter mechanism by using reflective cues (Multimedia Appendix 6), from early findings of an ongoing qualitative evaluation for this study, an alternative model using elaborative cues on why the choices of the health care providers were wrong would arguably enhance internalization of the learning content.

The high drop-off rate with each subsequent round of play despite a lack of maximization of the learning gains (Multimedia Appendices 14-16) was disconcerting and limited the assessment of learners’ skill mastery over time. The high drop-off rate might imply that (1) effort regulation and motivation for self-directed learning using LIFE is very low, (2) health care providers self-assessed learning needs lead them to believe that they have attained content mastery and thus are no longer in need of this training, or (3) other factors that are external to this experiment affect the use of the LIFE app (eg, uninstalling the LIFE app to make more space for other apps on the learner’s smartphone). Qualitative interviews will be used to explore whether any of these explanations are plausible and how they can be leveraged to encourage low drop-off rates.

From our findings, a gamified smartphone-based alternative to the low-dose-high-frequency clinical training approach commonly used in low-income settings that employs a self-regulated learning approach offers significant learning gains. This is useful where face-to-face training is not possible, costs of training are a concern, and learners prefer flexible learning schedules. In addition, the spaced repetition of such a learning approach can be encouraged after a week has passed, with the encouragement differentiated based on a knowledge tracing approach that is informed by the clinical role and experience in addition to learner progression. This can be explored in future research.

### Study Limitations

In this randomized experiment, we used the standard Bayesian Knowledge Tracing (BKT) modeling approach, which is explained in detail in the Multimedia Appendix 2 and elsewhere [49], to determine the adaptive feedback threshold cutoffs. However, BKT has a set of problematic assumptions: it assumes that forgetting does not occur, the knowledge components (ie, quizzes in our case) are treated as being mutually independent, its typical implementation does not allow learners to have different learning rates, it assumes that all students have the same probability of knowing a particular skill at their first opportunity (Multimedia Appendix 4), and it suffers from the problem of multiple global maxima when trying to estimate model parameters [49,72]. In addition, we used learning data from 187 health care providers from the beta phase to train the model, which is a relatively small number. Together with the moderate predictive accuracy of the health care provider’s knowledge (Multimedia Appendix 2), it is highly probable that the prescription of the level of feedback might have been biased, thereby underestimating the effect of the intervention. However, this modeling approach was used because it is more easily embedded within the smartphone app and was able to function offline compared with other common alternatives [49,73].

From interviews of the physically accessible study participants, those who were from lower clinical cadres found the one-off phone data cost necessary for downloading the smartphone app as being too high. To mitigate against burdening participants with this extra personal cost due to participating in the study, they could request for reimbursement within a few weeks of using the smartphone app. This skewed the study participants numbers toward a specific country where the reimbursements were made available (Multimedia Appendix 1). Within these resource contexts, such costs play a role in generating study participants who might not be representative of those with the intention to participate, rather representing those with the “economic” ability to participate, which is difficult to mitigate against in a multicountry study. It is challenging to determine if the reimbursement favored any study arm because of the blinded participant allocation to the study arms coupled with a lack of linking participants (whose data were collected as anonymous at source) to reimbursement.

The risk of performance bias across arms was moderate given that allowing for the “real-world” use of the smartphone app in a self-directed manner would also make it difficult to comprehensively control for exposure to factors outside the intervention of interest. This is especially true where some of the participants were in the same peer groups, hospitals, or social circles and might have collaboratively used the smartphone app. Although randomization allocation did not result in an equal number of participants in the study arms, from our post-hoc analysis (Table 1; Multimedia Appendices 10 and 11), we do not believe that this biased this experiment’s findings.

### Conclusions

This study set out to evaluate the effect of adaptive feedback within a smartphone-based serious game on the learning gains of health care providers from low-income countries. From 247 health care providers, the effect on learning gain was found to be g=0.09 (95% CI −0.31 to 0.46; *P*=.47). When subject-treatment interaction and differential time effect were controlled for, the effect of the adaptive feedback on learning gains increased significantly to 0.644 (95% CI 0.347 to 0.941; *P*<.001). From our findings, we suggest that linking the level of adaptive feedback provided to health care providers to how they space their learning and their clinical level might yield a larger intervention effect at both the group and individual learner levels. For the feedback content itself, as an alternative to using reflective hints on what the right answers might be, elaborating why the health care providers’ responses were wrong might enhance understanding of the learning content.

## Appendix

Multimedia Appendix 1 Participants breakdown by region.

Multimedia Appendix 2 More details on the intervention.

Multimedia Appendix 3 Bayesian Knowledge Tracing model parameters used.

Multimedia Appendix 4 Example of learning curves from the Bayesian Knowledge Tracing model for five different health care providers from the same learning task which are used to determined feedback level at each opportunity.

Multimedia Appendix 5 Adaptive feedback mechanism allocation within Life-Saving Instructions for Emergencies app.

Multimedia Appendix 6 Feedback content provided for the individual quizzes in Life-Saving Instructions for Emergencies smartphone app for Neonatal Resuscitation Scenario A Training.

Multimedia Appendix 7 Snapshots of the Life-Saving Instructions for Emergencies smartphone app.

Multimedia Appendix 8 Random allocation procedure.

Multimedia Appendix 9 Random allocation ratio based on time of day (the seed used for the generator).

Multimedia Appendix 10 Tests of whether differences in allocation rate between study arms resulted in non-equivalent Life-Saving Instructions for Emergencies app use for all study participants included.

Multimedia Appendix 11 Tests of whether dropout rate is different between study arms using baseline characteristics of study participants where demographic data are available.

Multimedia Appendix 12 Learning spacing between first two sessions, n (%).

Multimedia Appendix 13 Multicollinearity tests for the learning gain outcome.

Multimedia Appendix 14 Learners in each study arm at each learning iteration.

Multimedia Appendix 15 Change of student learning gains over learning sessions by study arm.

Multimedia Appendix 16 Change of student learning gains over successive learning sessions by spacing options.

Multimedia Appendix 17 CONSORT-EHEALTH checklist (V 1.6.1).

## Abbreviations

BKT: Bayesian Knowledge Tracing
ETAT+: Emergency Triage, Assessment, and Treatment Plus admission care training
KEMRI: Kenya Medical Research Institute
LIFE: Life-Saving Instructions for Emergencies
SRL: self-regulating learning
SSA: sub-Saharan Africa
