# Supplementary material for: Evaluation of Adaptive Feedback in a Smartphone-Based Game on Health Care Providers’ Learning Gain: Randomized Controlled Trial
Source: J Med Internet Res. 2020 Jul 6;22(7):e17100. doi: 10.2196/17100 (PMC7380991; doi:10.2196/17100)
Supplement: Multimedia Appendix 1 [file jmir_v22i7e17100_app1.docx]

| Multimedia Appendix 1: Participants breakdown by region | | | |
| --- | --- | --- | --- |
|  | **Continental Sub-Regions**  **(Developing Countries only)** | **Count (N)** | **Percentage (%)** |
| 1 | Sub-Saharan Africa* | 363 | 69.41 |
| 2 | South-Eastern Asia | 44 | 8.41 |
| 3 | Latin America and the Caribbean | 38 | 7.27 |
| 4 | Western Asia | 37 | 7.07 |
| 5 | Missing** | 23 | 4.4 |
| 6 | Eastern Asia | 12 | 2.29 |
| 7 | Northern Africa | 5 | 0.96 |
| *Note: *299 of these are Kenya participants, representing 52% of all recruited participants. **Even though LIFE’s requested Android permissions allow for collection of geographic regions of learner, there are cases where the mobile telecommunications provider did not transmit this information.* | | | |
