# Supplementary material for: Evaluation of Adaptive Feedback in a Smartphone-Based Game on Health Care Providers’ Learning Gain: Randomized Controlled Trial
Source: J Med Internet Res. 2020 Jul 6;22(7):e17100. doi: 10.2196/17100 (PMC7380991; doi:10.2196/17100)
Supplement: Multimedia Appendix 2 [file jmir_v22i7e17100_app2.docx]

# **APPENDIX**

## **Multimedia Appendix 2: More details on the intervention**

LIFE smartphone application evolves scenario-based teaching where the components being assessed emphasise the tenets of paediatric critical care with early recognition of children who need immediate care. Consequently, it follows a specific conjunctive ordering of clinical care-giving algorithms with each learning task being timed. Each quiz is a learning task representing a unique Knowledge Component (KC) step that addresses a learning “…concept, principle, fact, skill, schema, production rule, misconception…" [71] within the clinical care-giving algorithm.

The learner starts a scenario which provides some background information to care-giving scenario, and on each learning task, must provide input either through multiple choice questions, selection of items necessary for the learning task, or performing on-screen interactive tasks (e.g. navigating to equipment, switching on machines etc.) (Multimedia Appendix 3). On each incorrect attempt by the learner, feedback is provided with the option of more information and the learner must repeat until they successfully respond to the question before being allowed by the smartphone application to proceed. The end of the scenario is signalled by a crying baby indicating that the baby is now breathing, with a breakdown of scores by quiz provided. Each attempt, including the first attempt at a task in the current session, is timed. The learner has control over how long to contemplate over the question before attempting to answer it. Performance is calculated in the current learning session as the total score from all quiz attempted where the learner was correct on first try, without any feedback or help.

What the learner does during the learning sessions is captured by the number of quizzes attempted, the number of tries per quiz, the amount of time they spend on each quiz, the level of feedback provided with each try, and final performance score. LIFE’s design emulates the low-dose high-frequency (LDHF) training model to encourage repeated refreshing of emergency care knowledge but without any face-to-face facilitation or classroom learning. In low resource settings, this model -although typically face-to-face, has been shown to be effective for clinical care training in SSA [72]. In this study, only scenario A was used as scenario B and C were not ready for release when the study started. They were introduced later during the experiment as an Android application update, but scenario A remained the same throughout the trial. This did not interfere with the progress of the learners as either way, they had to attain a perfect score in Scenario A for Scenario B and C to be unlocked.

## **Bayesian Knowledge Tracing (BKT) models and adaptive feedback mechanism**

The detailed explanation of how the standard BKT modelling approach used in determining feedback level works is provided elsewhere [46]. The procedure of how to fit a BKT model is provided in detail in this GitHub Repository [73]. In summary, the fitted BKT model used Gradient Descent optimisation algorithm for the loss function and had a predictive accuracy of 0.6431 (Log likelihood= 3955.25, AIC = 8006.50, BIC=8329.73, RMSE=0.4708). The model values used for tracing individual healthcare provider’s knowledge are provided in Multimedia Appendix 4 and include: (a) probability of knowing a skill a priori, (b) probability of the learner transitioning from not known to known state after an opportunity to apply it, (c) probability to make mistake when applying a skill, and (d) probability of applying a skill correctly when you do not know it. After each quiz attempt, the predicted probability that the healthcare provider had learnt the concept was recalculated based on values in Multimedia Appendix 4. This was quiz specific and varied by healthcare provider based on their cumulative success rate on that specific quiz. The is illustrated in Multimedia Appendix 5 using five healthcare providers.

Probability of getting the next attempt correct (explained in detail elsewhere [46]) was used as the decision criteria for deciding level of feedback to provide. Probability thresholds of ***<0.4***, **0.4 to 0.7** and **>0.7** were used for detailed, reflective and minimal feedback respectively for the experiment arm. The provision of feedback followed the mechanism illustrated in Multimedia Appendix 6. The content of the levels of feedback highlighted in Multimedia Appendix 6 are detailed in Multimedia Appendix 7. Level 1 feedback was only available to those in the experiment group with the BKT mechanism explained in detail elsewhere [46].

## **Randomisation algorithm**

Multimedia Appendix 8 outlines how randomisation was happening on the Android Smartphones. Because the random generator function (C++ ***rand*** function) uses a range of 4 values only i.e. [0,3], together with its unreliable performance on different computing devices [58, 62], it is highly likely this was what contributed to the disparity in the allocation ratio illustrated in the Multimedia Appendix 9. This is compounded by a lack of an enforceable blocking mechanism based on how we implemented randomisation. This in turn made random allocation skewed towards the control group. However, it did not render the groups non-comparable for this experimental setup (Multimedia Appendix 10, Multimedia Appendix 11).

## **Outcome calculation**

### **Primary outcome calculation: Effect of adaptive feedback on learning gains across study arms**

$$Effect (Morris g)= C_{pp}*\left( \frac{\left( M_{post,T}- M_{pre,T} \right)-\left( M_{post,C}- M_{pre,C} \right)}{{SD}_{pre+post}} \right) (1)$$

where the pooled standard deviation is defined as

$${SD}_{pre+post}= \sqrt{\frac{\left( n_{T}-1 \right){SD}_{pre,T}^{2}+\left( n_{C}-1 \right){SD}_{pre,C}^{2}+\left( n_{T}-1 \right){SD}_{post,T}^{2}+(n_{C}-1){SD}_{post,C}^{2}}{2\left( n_{T}+n_{C}-2 \right)}} (2)$$

and the bias adjustment is provided by the formula:

$$C_{pp}=1- \frac{3}{4\left( 2n_{T}+2n_{C}-4 \right)-1} (3)$$

and

*Sample size of treatment group:* $n_{T}$

*Pre-test mean for treatment group:* $M_{pre,T}$

*Post-test mean for treatment group:* $M_{post,T}$

*Standard deviation of means for pre-test in treatment group:* ${SD}_{pre,T}$

*Standard deviation of means for post-test in treatment group:* ${SD}_{post,T}$

*Sample size of control group:* $n_{C}$

*Pre-test mean for control group:* $M_{pre,C}$

*Post-test mean for control group:* $M_{post,C}$

*Standard deviation of means for pre-test in control group:* ${SD}_{pre,C}$

*Standard deviation of means for post-test in control group:* ${SD}_{post,C}$

### **Secondary outcome calculation: Effect of adaptive feedback on individual learning gains**

$$Individualised normalised learning gain =\left\{ \begin{matrix} \frac{post-pre}{100-pre} & post>pre \\ drop & post=pre=100=0 \\ 0 & post=pre \\ \frac{post-pre}{pre} & post<pre \end{matrix} \right. (4)$$
