# Supplementary material for: Evaluation of Adaptive Feedback in a Smartphone-Based Game on Health Care Providers’ Learning Gain: Randomized Controlled Trial
Source: J Med Internet Res. 2020 Jul 6;22(7):e17100. doi: 10.2196/17100 (PMC7380991; doi:10.2196/17100)
Supplement: Multimedia Appendix 3 [file jmir_v22i7e17100_app3.docx]

| Multimedia Appendix 4: Bayesian Knowledge Tracing model parameters used | | | | |
| --- | --- | --- | --- | --- |
| Quiz | **Prior (a)** | **Transition (b)** | **Slip (c)** | **Guess (d)** |
| 1 | 0.5172 | 0.2526 | 0.2051 | 0.1254 |
| 2 | 0.6113 | 0.2092 | 0.2778 | 0.0982 |
| 3 | 0.1675 | 0.4096 | 0.2251 | 0.2949 |
| 4 | 0.5199 | 0.3569 | 0.2296 | 0.1931 |
| 5 | 0.5647 | 0.266 | 0.2426 | 0.1317 |
| 7 | 0.4591 | 0.419 | 0.1426 | 0.2393 |
| 9 | 0.5152 | 0.4655 | 0.1149 | 0.268 |
| 10 | 0.5283 | 0.3712 | 0.2374 | 0.1954 |
| 12 | 0.4586 | 0.3819 | 0.0982 | 0.2041 |
| 13 | 0.3441 | 0.3594 | 0.1003 | 0.2731 |
| *Note*: No Reflective feedback for was provided for Quiz 6, 8, and 11 because of ongoing changes to these quizzes during the study period. | | | | |
