# Supplementary figures and images for: Evaluation of Adaptive Feedback in a Smartphone-Based Game on Health Care Providers’ Learning Gain: Randomized Controlled Trial
Source: J Med Internet Res. 2020 Jul 6;22(7):e17100. doi: 10.2196/17100 (PMC7380991; doi:10.2196/17100)

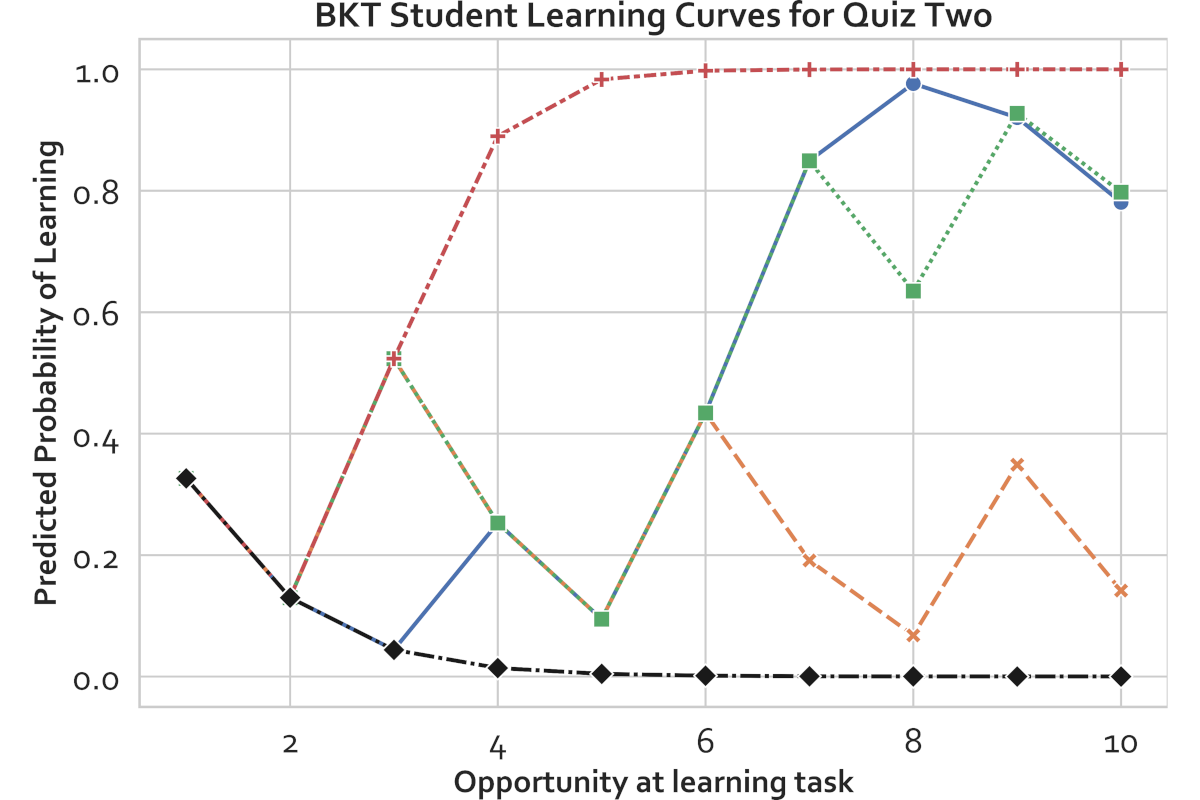

Supplement: Multimedia Appendix 4 [file jmir_v22i7e17100_app4.png]

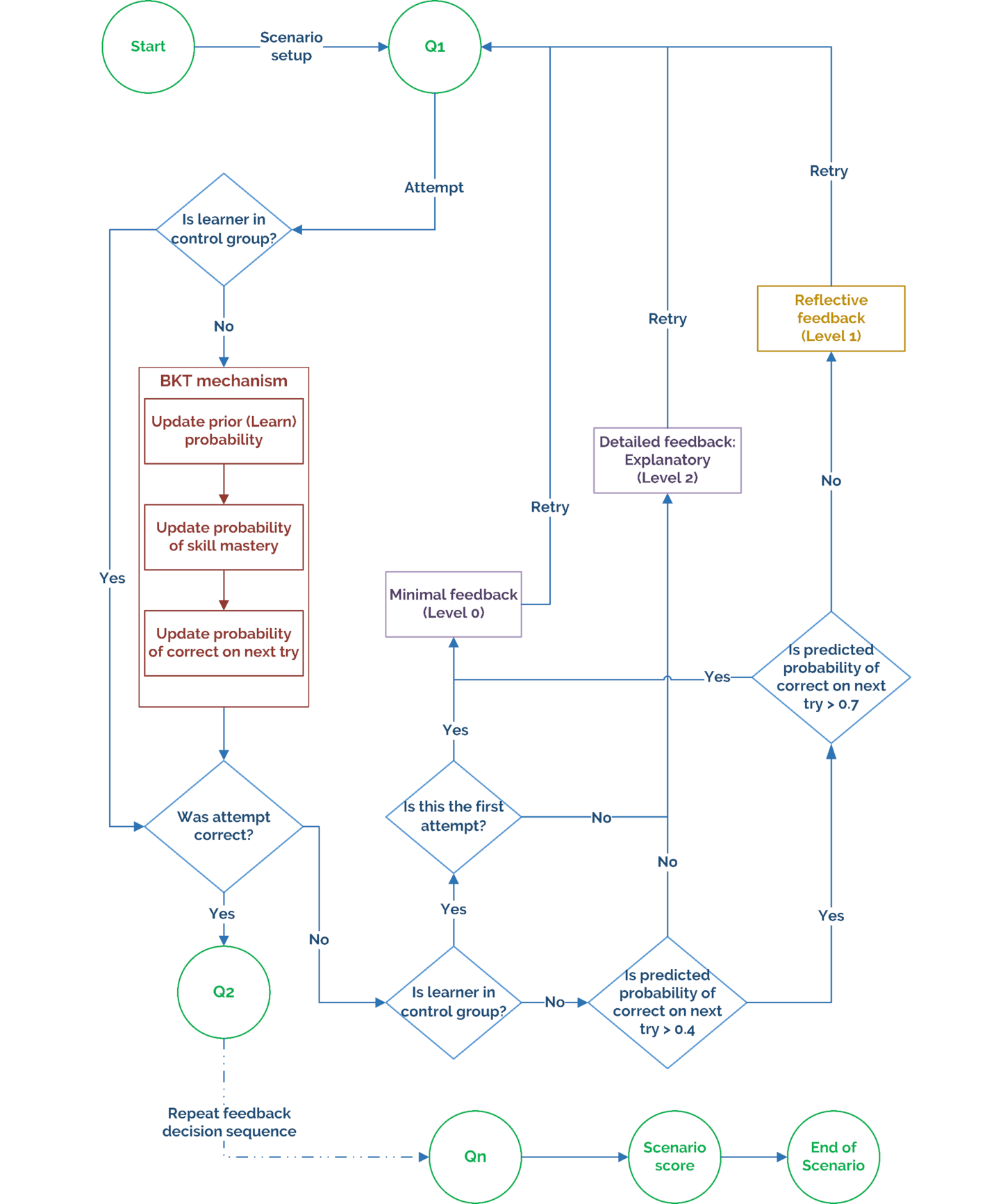

Supplement: Multimedia Appendix 5 [file jmir_v22i7e17100_app5.png]

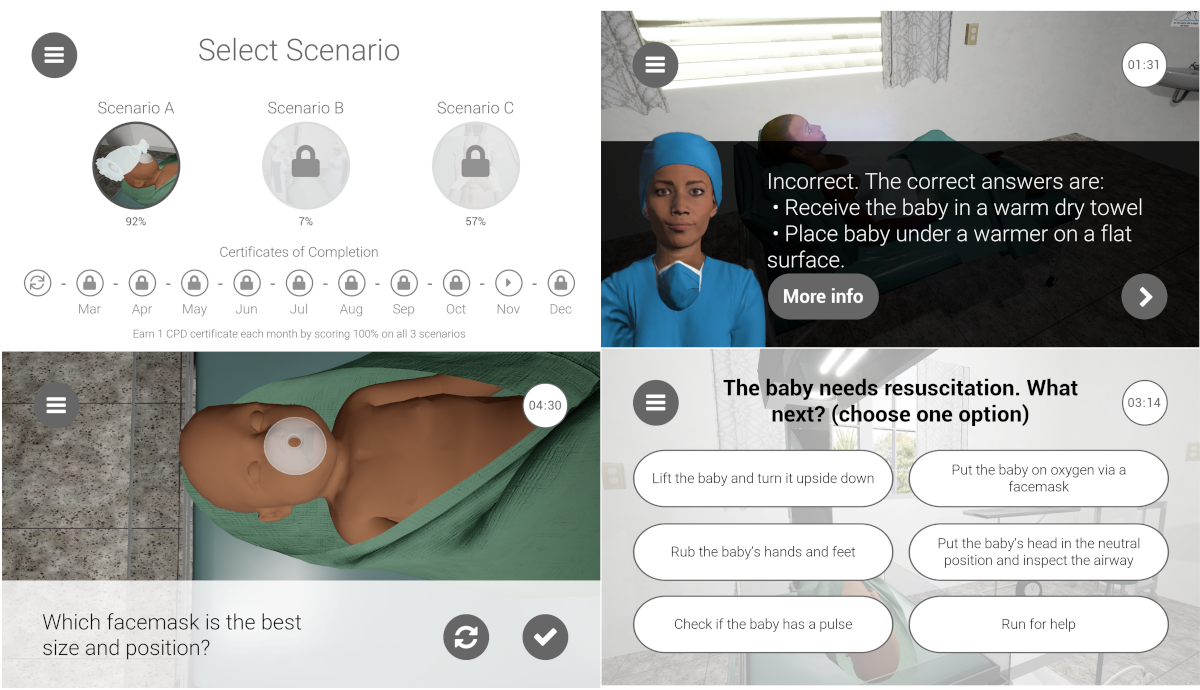

Supplement: Multimedia Appendix 7 [file jmir_v22i7e17100_app7.png]

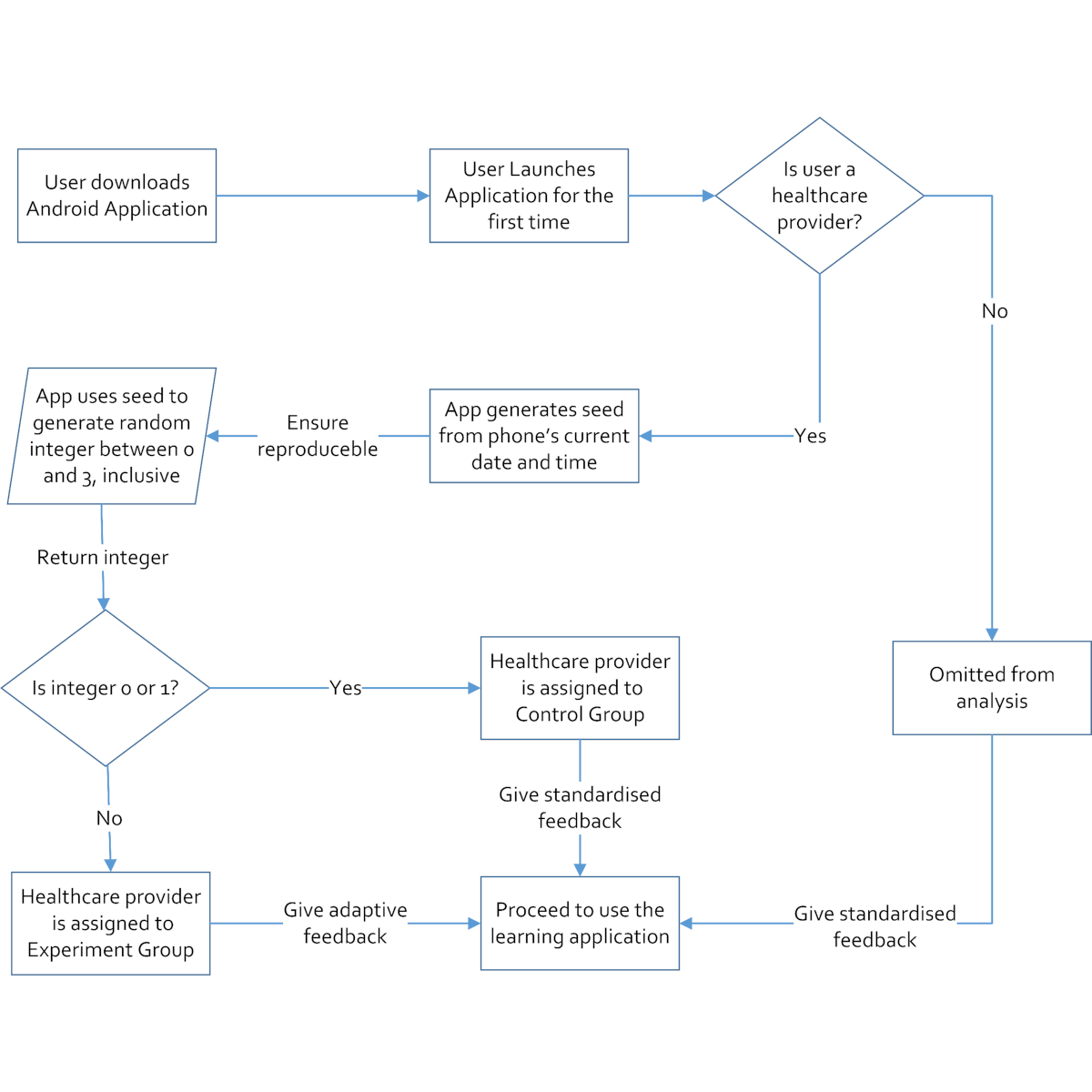

Supplement: Multimedia Appendix 8 [file jmir_v22i7e17100_app8.png]

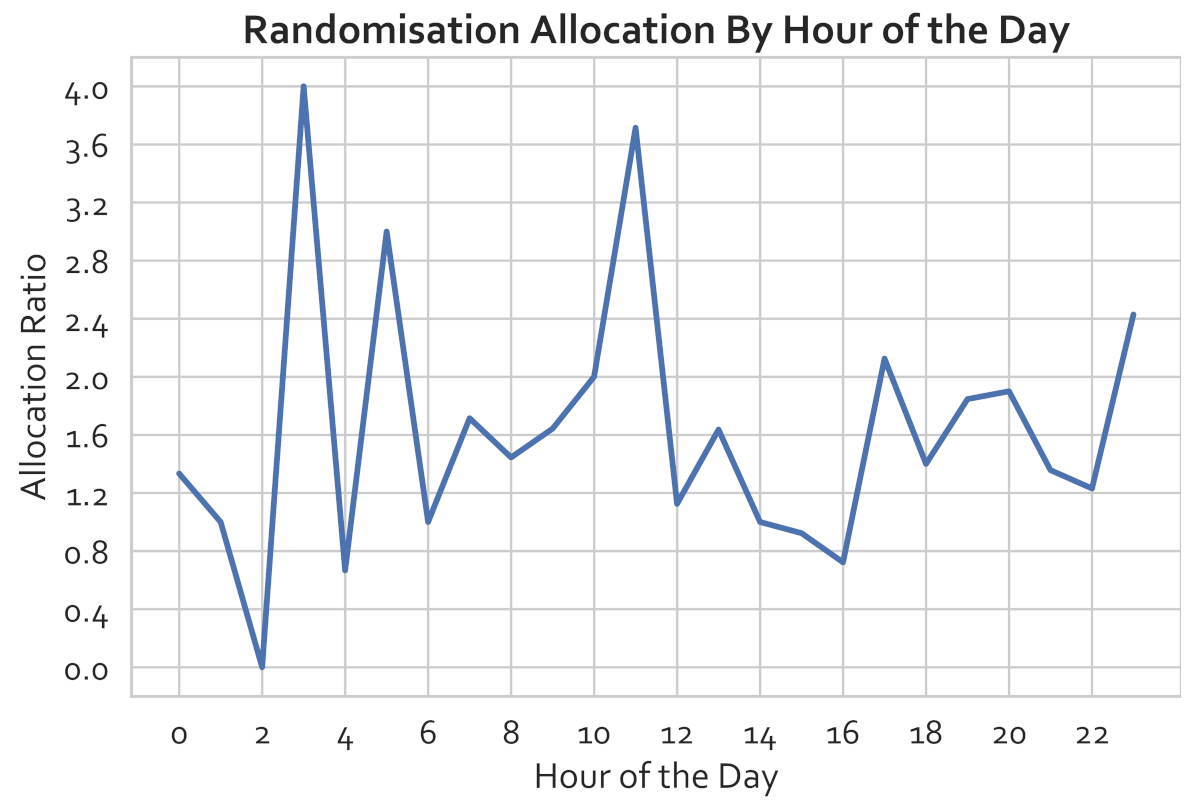

Supplement: Multimedia Appendix 9 [file jmir_v22i7e17100_app9.png]

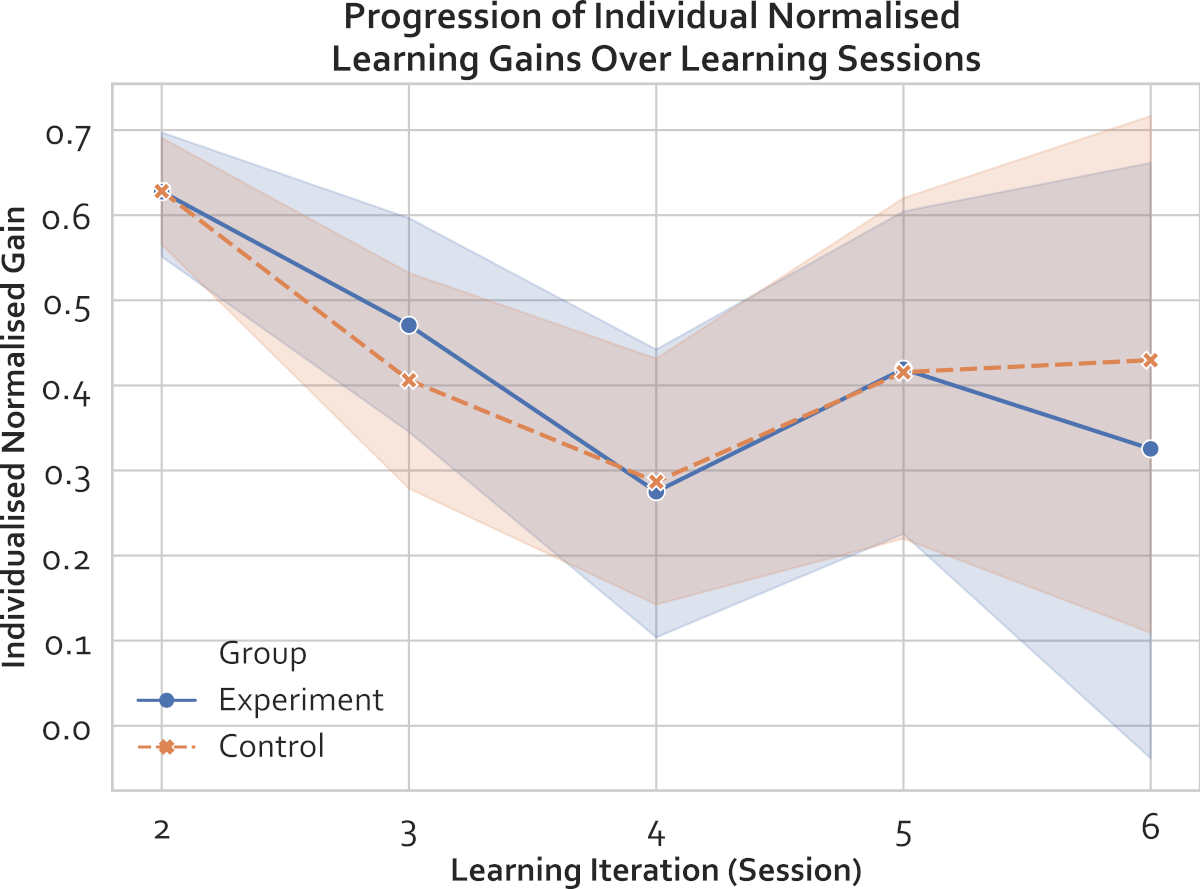

Supplement: Multimedia Appendix 15 [file jmir_v22i7e17100_app15.png]

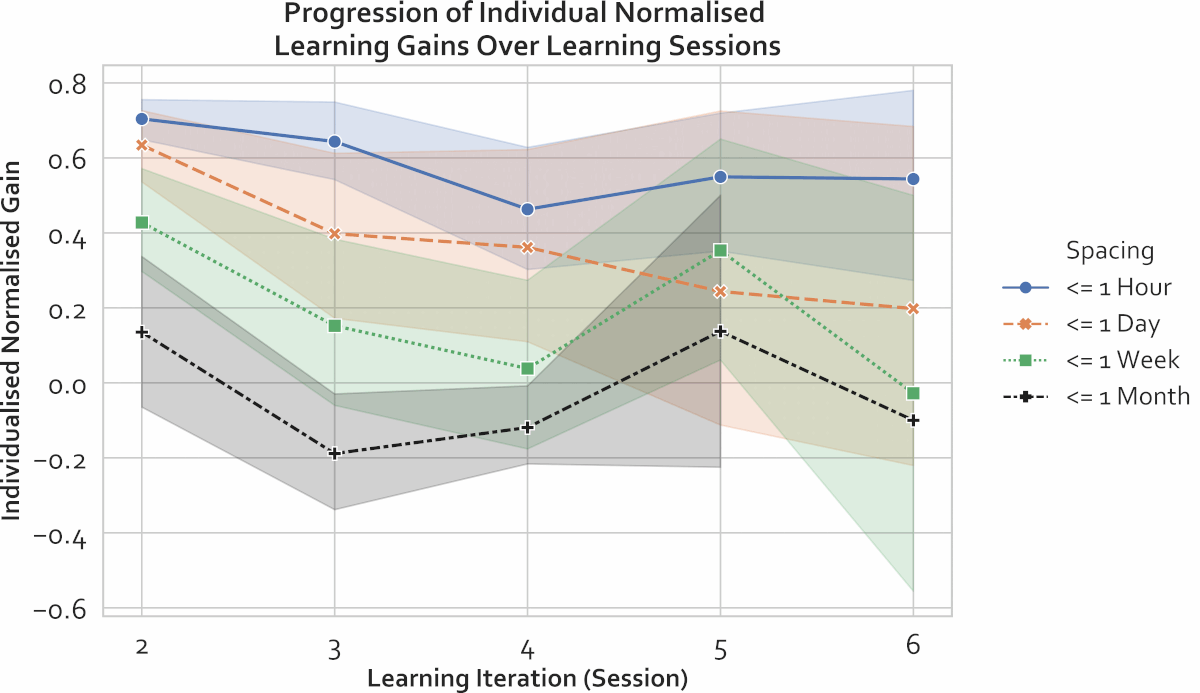

Supplement: Multimedia Appendix 16 [file jmir_v22i7e17100_app16.png]
