# Supplementary material for: Evaluation of Adaptive Feedback in a Smartphone-Based Game on Health Care Providers’ Learning Gain: Randomized Controlled Trial
Source: J Med Internet Res. 2020 Jul 6;22(7):e17100. doi: 10.2196/17100 (PMC7380991; doi:10.2196/17100)
Supplement: Multimedia Appendix 6 [file jmir_v22i7e17100_app6.docx]

| Multimedia Appendix 7: Feedback content provided in LIFE smartphone application for Neonatal Resuscitation Scenario A Training for the individual quizzes | | | |
| --- | --- | --- | --- |
| Feedback  Quiz | Level 0 (Minimal) | Level 1 (Reflective) | Level 2 (Detailed) |
| Quiz 1 | No, that is not correct. Try Again. | [**Both/One/None**] of these pieces of information are incorrect in directly affecting the recommended sequence of actions to resuscitate a new-born baby. | Incorrect. The two key questions are: • Is there any meconium in the liquor? • Is there anyone else who can assist me?  Both these pieces of information directly affect the recommended sequence of actions to resuscitate a new-born baby. The other questions certainly affect the health of mother and / or baby and will be useful to know at some stage, but they are not vital for the first few minutes of neonatal resuscitation. |
| Q2 | No, that is not correct. Try Again. | **[X]** of these pieces were incorrect.  Of the selected, **[Y]** are not necessary | This selection was incorrect. In addition to the resuscitaire (a flat, warm surface) you need the following:  • warm towels • a bag-valve device • small facemasks (size 0 or 1)  • a suction machine • a wide-bore sucker • a clock  • stethoscope  These are essential for basic resuscitation of the new-born. |
| Quiz 3 | These actions are not appropriate at this stage. Try again. | **[Both/One]** of the selected choices are not appropriate | Incorrect. The correct answers are:  • Receive the baby in a warm dry towel  • Place baby under a warmer on a flat surface. |
| Quiz 4 | These actions are not appropriate at this stage. Try again. | **[Both/One]** of these actions are not appropriate at this stage. Try again. | Incorrect. The correct questions are:  • Dry the baby all over and stimulate by rubbing its back for 20 seconds  • Wrap the baby in a warm dry towel |
| Quiz 5 | These actions are not appropriate at this stage. Try again. | **[All/Some/One]** of the selected actions are not appropriate at this stage | Incorrect. The correct questions are:  • Is the baby crying?  • Is the baby moving?  • Is the baby pink rather than pale? |
| Quiz 6 | Try again |  | Correct Answer: • Shout for help |
| Quiz 7 | No, that is not correct. Try again. What will you do next? | Here's a hint: neither **[user answer]** nor **Y** is correct. Try again. | Incorrect. The most important thing to do once you have decided the baby needs resuscitation is to put the baby’s head in the neutral position. |
| Quiz 8 | That is not the best head position for the airway. Try again |  | The correct head position is shown here. Try again. |
| Quiz 9 | No, that is not correct. Try again. | Here's a hint: neither **[user answer]** nor **Y** is correct. Try again. | Incorrect. It is important now the airway is open to recheck for breathing. Try again. |
| Quiz 10 | No, that is not correct. Try again. | Here's a hint: neither **[user answer]** nor **Y** is correct. Try again. | If the baby is not breathing, the priority is to ventilate using a bag valve mask. Try again. |
| Quiz 11 | That is incorrect. This mask is not the right size for this baby. Try again |  | This is incorrect. The correct facemask is shown here. The facemask should fit over the nose and mouth of the neonate and make a good seal with the face and chin, without covering all or part of the eyes. The shape is less important than the correct size and either circular or triangular masks are fine. Generally, a size 0 or size 1 mask will be the best fit for a new-born. It is important not only to have the correct size but also to position it well, so that air can be pushed into the lungs through the open airway and not leak out around the sides between the mask and the face. |
| Quiz 12 | That is not correct. Try again. | **[All/Some/One]** of these options are not appropriate. Try again. | That is not correct. The priority is to inflate the lungs with air. To do this you need to maintain an open airway using the neutral head position, give 30 breaths per minute and see the chest rise and fall when you squeeze the bag. Other actions will delay this life-saving manoeuvre. |
| Quiz 13 | No, that is not correct. Try again. | Here's a hint: neither **[user answer]** nor **Y** is correct. Try again. | That is not correct. You should now check for a pulse to identify whether the baby has an adequate cardiac output. This is not done earlier because the most common problem a new-born has is failure to breathe. |
| *Note:* No Reflective feedback for was provided for Quiz 6, 8, and 11 because of ongoing changes to these quizzes during the study period. | | | |
