# Supplementary material for: Evaluation of Adaptive Feedback in a Smartphone-Based Game on Health Care Providers’ Learning Gain: Randomized Controlled Trial
Source: J Med Internet Res. 2020 Jul 6;22(7):e17100. doi: 10.2196/17100 (PMC7380991; doi:10.2196/17100)
Supplement: Multimedia Appendix 10 [file jmir_v22i7e17100_app10.docx]

| Multimedia Appendix 10: Tests of whether differences in allocation rate between study arms resulted in non-equivalent LIFE app use for all study participants included | | | | | | |
| --- | --- | --- | --- | --- | --- | --- |
| Indicator | All eligible participants  (N=572) | | | Participants who reached endpoint  (n=247) | | |
|  | Control Arm  (n=344) | Experiment Arm (n=228) | P-value* | Control Arm  (n=148) | Experiment Arm (n=99) | P-value* |
|  | *Mean (SD)* | *Mean (SD)* |  | *Mean (SD)* | *Mean (SD)* |  |
| Number of sessions started | 2.57 (2.54) | 2.75 (2.51) | 0.41 | 4.22 (3.03) | 4.61 (2.87) | 0.31 |
| Number of sessions completed | 2.31 (1.66) | 2.54 (1.83) | 0.21 | 3.17 (1.66) | 3.51 (1.85) | 0.15 |
| Time taken per session (in seconds) | 205.83 (141.98) | 189.89 (124.64) | 0.16 | 198.54 (91.13) | 201.33 (96.54) | 0.82 |
| Questions attempted per session | 7.31 (3.33) | 6.9 (3.48) | 0.16 | 9.15 (1.28) | 9.05 (1.24) | 0.54 |
| Time per learning task (in seconds) | 14.79 (14.82) | 15.44 (17.11) | < 0.01 | 14.15 (14.44) | 15.53 (17.76) | <0.001 |
| Feedback messages per learning session | 4.99 (6.71) | 4.83 (6.30) | 0.64 | 4.27 (6.55) | 4.12 (5.30) | 0.70 |
| Performance** | 61.94 (24.79) | 62.15 (21.03) | 0.92 | 71.35 (15.9) | 68.94 (14.86) | 0.23 |
| Note: *From difference in means t-test between the two study arms. **Calculated as the percentage of quiz correct on first try out of all quizzes attempted. | | | | | | |
