# Supplementary material for: Evaluation of Adaptive Feedback in a Smartphone-Based Game on Health Care Providers’ Learning Gain: Randomized Controlled Trial
Source: J Med Internet Res. 2020 Jul 6;22(7):e17100. doi: 10.2196/17100 (PMC7380991; doi:10.2196/17100)
Supplement: Multimedia Appendix 11 [file jmir_v22i7e17100_app11.docx]

| Multimedia Appendix 11: Tests of whether dropout rate is different between study arms using baseline characteristics of study participants where demographic data are available | | | | | | | |
| --- | --- | --- | --- | --- | --- | --- | --- |
| Indicator | Healthcare providers who did not  reach study’s primary endpoint (n=40) | | | Healthcare providers who reached  the study's primary endpoint (n=135) | | | Attrition Bias Check |
|  | Control (n=19) | Experiment (n=21) | P-value* | Control  (n=84) | Experiment  (n=51) | P-value* |  |
|  | Mean (SD) | |  | Mean (SD) | |  | **P-value**** |
| Age (years) | 32.95 (8.26) | 29.95 (6.5) | 0.188 | 30.81 (8.42) | 29.18 (7.24) | 0.235 | 0.401 |
| Experience (years) | 8.84 (7.05) | 6.4 (6.59) | 0.326 | 6.83 (6.66) | 6.46 (8.33) | 0.793 | 0.471 |
| Sessions Started | 2.16 (2.46) | 1.48 (0.6) | 0.252 | 4.32 (3.00) | 5.35 (3.39) | 0.077 | N/A |
|  | | | | | | | |
|  | **N (%)** | | **P-value^ǂ^** | **N (%)** | | **P-value^ǂ^** | **P-value^ǂǂ^** |
| *Clinical Cadre* | | | | | | | |
| Doctor | 6 (31.58%) | 8 (38.1%) | 0.333 | 31 (36.9%) | 22 (43.14%) | 0.236 | 0.148 |
| Clinical Officer | 3 (15.79%) | 5 (23.81%) | 0.263 | 14 (16.67%) | 5 (9.8%) | 0.867 | **0.038** |
| Nurse | 9 (47.37%) | 7 (33.33%) | 0.817 | 31 (36.9%) | 21 (41.18%) | 0.31 | 0.405 |
| Other | 1 (5.26%) | 1 (4.76%) | 0.529 | 8 (9.52%) | 3 (5.88%) | 0.773 | 0.261 |
|  |  |  |  |  |  |  |  |
| *Clinical Training Level (whether completed general/speciality training and professionally registered)* | | | | | | |  |
| Specialised | 4 (21.05%) | 5 (23.81%) | 0.417 | 19 (22.62%) | 6 (11.76%) | 0.942 | **0.041** |
| General Officer | 9 (47.37%) | 8 (38.1%) | 0.723 | 29 (34.52%) | 21 (41.18%) | 0.219 | 0.358 |
| Intern | 1 (5.26%) | 1 (4.76%) | 0.529 | 8 (10.71%) | 6 (11.76%) | 0.339 | 0.394 |
| Student | 5 (26.32%) | 7 (33.33%) | 0.315 | 28 (33.33%) | 18 (35.29%) | 0.408 | 0.116 |
|  |  |  |  |  |  |  |  |
| Done ETAT+? | 11 (57.89%) | 14 (66.67%) | 0.283 | 49 (58.33%) | 33 (64.71%) | 0.231 | 0.082 |
| Note: *P-value from difference in means between study arms within dropout category. **P-value from test in difference in means between due to dropout. ^ǂ^ P-value from a difference in proportions test between study arms within progress category.  ^ǂǂ^P-value from test of difference in dropout rate between the intervention groups. | | | | | | | |
