# Supplementary material for: Evaluation of Adaptive Feedback in a Smartphone-Based Game on Health Care Providers’ Learning Gain: Randomized Controlled Trial
Source: J Med Internet Res. 2020 Jul 6;22(7):e17100. doi: 10.2196/17100 (PMC7380991; doi:10.2196/17100)
Supplement: Multimedia Appendix 12 [file jmir_v22i7e17100_app12.docx]

| Multimedia Appendix 12: Learning spacing between first two sessions, n (%) | | | | | | |
| --- | --- | --- | --- | --- | --- | --- |
| Spacing Length | ≤**1 Hour** | ≤**1 Day** | ≤**1 Week** | ≤**1 Month** | **>1 Month** | **All** |
| Control | 97 (39) | 30 (12) | 11(4) | 7 (3) | 3(1) | 148(59.9) |
| Experiment | 66 (27) | 17 (7) | 8(3) | 7 (3) | 1(0) | 99(40) |
| All | 163 (66.0) | 47 (19) | 19(8) | 14 (6) | 4(2) | 247 |
