# Supplementary material for: Evaluation of Adaptive Feedback in a Smartphone-Based Game on Health Care Providers’ Learning Gain: Randomized Controlled Trial
Source: J Med Internet Res. 2020 Jul 6;22(7):e17100. doi: 10.2196/17100 (PMC7380991; doi:10.2196/17100)
Supplement: Multimedia Appendix 13 [file jmir_v22i7e17100_app13.docx]

| Multimedia Appendix 13: Multicollinearity tests for the learning gain outcome | | | | |
| --- | --- | --- | --- | --- |
|  | **Time** | **Help** **Ratio** | **Age** | **Experience** |
| Variance Inflation  Factor (VIF) | 1.041328 | 1.018684 | 1.025009 | 1.046533 |
| Note: *With variable correlations (VIF) of less than 1.05, the regression results from*  *secondary analyses (Table 3) are considered very reliable.* | | | | |
