# Supplementary material for: Evaluation of Adaptive Feedback in a Smartphone-Based Game on Health Care Providers’ Learning Gain: Randomized Controlled Trial
Source: J Med Internet Res. 2020 Jul 6;22(7):e17100. doi: 10.2196/17100 (PMC7380991; doi:10.2196/17100)
Supplement: Multimedia Appendix 14 [file jmir_v22i7e17100_app14.docx]

| Multimedia Appendix 14: Learners in study arms over each iteration | | | | | | | |
| --- | --- | --- | --- | --- | --- | --- | --- |
| ^Iteration^  _Group_ | **1** | **2** | **3** | **4** | **5** | **6** | **7**+ |
| Control | 242 (25.2%) | 148 (15.4%) | 75 (8%) | 40 (4%) | 27 (3%) | 12 (1%) | 17 (2%) |
| Experiment | 158 (16.4%) | 99 (10%) | 59 (6%) | 38 (4%) | 22 (2%) | 13 (1%) | 12 (1%) |
| All | 400 (41.6%) | 247 (25.7%) | 134 (13.9%) | 78 (8%) | 49 (5%) | 25 (3%) | 29 (3%) |
